# Melatonin Supplementation, a Strategy to Prevent Neurological Diseases through Maintaining Integrity of Blood Brain Barrier in Old People

**DOI:** 10.3389/fnagi.2017.00165

**Published:** 2017-05-24

**Authors:** Wen-Cao Liu, Xiaona Wang, Xinyu Zhang, Xi Chen, Xinchun Jin

**Affiliations:** ^1^Department of Emergency, Shanxi Provincial People’s HospitalTaiyuan, China; ^2^Jiangsu Key Laboratory of Translational Research and Therapy for Neuro-Psycho-Diseases and Institute of Neuroscience, Department of Neurology, the Second Affiliated Hospital of Soochow UniversitySuzhou, China; ^3^School of Pharmacy, Key Laboratory of Molecular Pharmacology and Drug Evaluation (Yantai University), Ministry of Education, Yantai UniversityYantai, China; ^4^Department of Core Facility, the People’s Hospital of Baoan ShenzhenShenzhen, China

**Keywords:** melatonin, blood brain barrier, neurological diseases, old people, lipopolysaccharide

## Abstract

Blood brain barrier (BBB) plays a crucial role in maintaining homeostasis of microenvironment that is essential to neural function of the central nervous system (CNS). When facing various extrinsic or intrinsic stimuli, BBB is damaged which is an early event in pathogenesis of a variety of neurological diseases in old patients including acute and chronic cerebral ischemia, Alzheimer’s disease and etc. Treatments that could maintain the integrity of BBB may prevent neurological diseases following various stimuli. Old people often face a common stress of sepsis, during which lipopolysaccharide (LPS) is released into circulation and the integrity of BBB is damaged. Of note, there is a significant decrease of melatonin level in old people and animal. Melatonin has been shown to preserves BBB integrity and permeability via a variety of pathways: inhibition of matrix metalloproteinase-9 (MMP-9), inhibition of NADPH oxidase-2, and impact on silent information regulator 1 (SIRT1) and nucleotide-binding oligomerization domain-like receptor family pyrin domain-containing 3 (NLRP3) inflammasome. More important, a recent study showed that melatonin supplementation alleviates LPS-induced BBB damage in old mice through activating AMP-activated protein kinase (AMPK) and inhibiting gp91^phox^, suggesting that melatonin supplementation may help prevent neurological diseases through maintaining the integrity of BBB in old people.

## Introduction

### The Blood Brain Barrier Damage and Neurological Diseases

**Figure 1:**
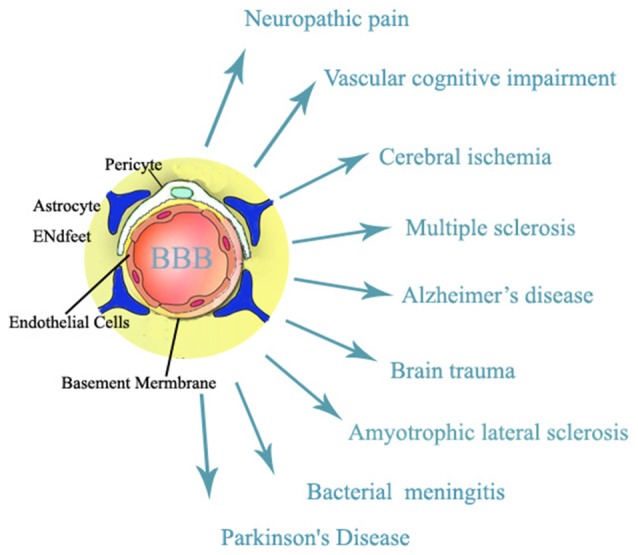
**Structure of blood brain barrier (BBB) which is related to neurological diseases**.

The blood brain barrier (BBB) is a regulated interface between the peripheral circulation and the central nervous system (CNS; Jin et al., 2014). BBB, which is composed of cerebral microvascular endothelial cells, neurons, astrocytes, pericytes and the extracellular matrix, plays a key role in maintaining homeostasis of microenvironment that is essential to neural function of the CNS (Hawkins and Davis, 2005). When facing various extrinsic or intrinsic stimuli (Weiss et al., 2009), BBB is damaged and BBB dysfunction is an early event in pathogenesis of a variety of neurological diseases in old patients including vascular cognitive impairment, amyotrophic lateral sclerosis, Alzheimer’s disease, neuropathic pain, brain trauma, acute and chronic cerebral ischemia, multiple sclerosis, and brain infections (Rosenberg, 2012; please see Figure 1). Treatments that could maintain the integrity of BBB will have important roles in preventing stimuli-produced neurological diseases.

The tight junction proteins (TJPs), composed of occludin, claudin and zo-1, are key components of the BBB (Hawkins and Davis, 2005). It seals the interendothelial cleft forming a continuous blood vessel, leads to high endothelial electrical resistance, and allows low paracellular permeability of water-soluble substances from the blood into brain parenchyma (Stamatovic et al., 2008). Free radicals of oxygen and nitrogen and the proteases, matrix metalloproteinases (MMPs) and cyclooxgyenases, play key roles in the early and delayed BBB disruption as the neuroinflammatory response progresses (Liu and Rosenberg, 2005). During an injury, free radicals and proteases attacked the cell membranes and degraded the TJPs between endothelial cells and the integrity of BBB is damaged (Jin et al., 2013; Liu et al., 2016; Wang et al., 2016). It is worth of note, death of endothelial cells of microvessels is also a major contributor to the disruption of BBB integrity (Simard et al., 2007). Therefore, protective effect on intergrity of BBB should consider both death of endothelial cells of microvessels and degradation of TJPS.

### BBB Disruption Induced by Lipopolysaccharide (LPS)

Lipopolysaccharide (LPS) could produce neuroinflammation (Shi, 2015), promoting the generation of reactive oxygen species (ROS) in cerebral microvascular endothelial cells and BBB disruption (Seok et al., 2013). Worth of note, LPS has been shown to increase BBB permeability *in vitro* (Nonaka et al., 2004) and compromise BBB integrity in young (Ruiz-Valdepeñas et al., 2011; Zhou T. et al., 2014) and old mice (Wang et al., 2017). More interesting, LPS has been shown to induce BBB dysfunction via nicotinamide adenine dinucleotide phosphate (NADPH) oxidase-derived ROS (Liu et al., 2012; Zhao et al., 2014). NADPH oxidases, a major source of ROS generation in the brain, critically contributes to BBB disruption under various neurological disorders (Kahles et al., 2007). Of note, gp91^phox^ is the catalytic subunit of NADPH oxidase and BBB disruption is significantly reduced in gp91^phox^ knockout mice compared to wild-type mice after stroke (Kahles et al., 2007) and reduction of gp91^phox^ expression has shown protective effect against ischemia-induced brain injury and BBB damage (Liu et al., 2008, 2011). More importantly, Wang et al. (2017) showed that LPS increased gp91^phox^ expression in both endothelial cells and in old mice, suggesting that gp91^phox^ up-regulation may be an important mechanism responsible for LPS-induced BBB permeability increase in old mice.

### Relationship between Melatonin and Aging

Melatonin, which is produced mainly in the pineal gland, retina and the gastrointestinal tract, plays important roles in many physiological and biochemical functions (Bubenik and Konturek, 2011), such as acting as an anti-inflammatory and immunoregulating molecule as well as a circadian rhythm regulator (Manchester et al., 2015). Melatonin is a potent free radical scavenger, lack of melatonin may result in decreased antioxidant function in the old people which have significant influence not only on aging *per se* but also on the incidence or severity of age-related diseases (Karasek, 2004). In addition, oxygen radical detoxification processes was significantly decreased during aging and there was a obvious downregualtion in pineal biosynthetic activity in aging hamster (Bubenik and Konturek, 2011). More interesting, melatonin levels in serum and brain decline as a result of aging (Bubenik and Konturek, 2011; Hill et al., 2013). In addition, melatonin has been reported to regulate aging and neurodegeneration through energy metabolism, epigenetics, autophagy and circadian rhythm pathways (Jenwitheesuk et al., 2014).

### Beneficial Role of Melatonin in Sepsis

Sepsis is a systemic inflammatory response to infection that causes severe neurological complications (Zhao et al., 2015) and it is a common stress that old people often face (Martin et al., 2006), in which LPS is released into circulation (Shukla et al., 2014).

Melatonin has been shown to restore the mitochondrial production of ATP in septic mice (López et al., 2006a), block the septic response by disrupting connection of the nuclear factor kappa-light-chain-enhancer of activated B cells (NF-κB) with nucleotide-binding oligomerization domain-like receptor family pyrin domain-containing 3 (NLRP3) in mice (El Frargy et al., 2015) and improve survival in a zymosan A-induced rat model of sepsis/shock (Reynolds et al., 2003). In addition, melatonin has been shown to protect organs against sepis-inuduced injury. For example, melatonin improved cardiac mitochondria and survival rate in rat septic heart injury (Zhang et al., 2013) through inhibition of inducible nitric oxide synthase (iNOS) and preservation of neuronal nitric oxide synthase (nNOS; Ortiz et al., 2014) and attenuated sepsis-induced cardiac dysfunction via a PI3K/Akt-dependent mechanism (An et al., 2016). Furthermore, melatonin protected liver bioenergetics from sepsis-induced damage (Basile et al., 2004), modified cellular stress in the liver of septic mice by reducing ROS and increasing the unfolded protein response (Kleber et al., 2014), protected against sepsis-induced functional and biochemical changes in rat ileum and urinary bladder (Paskaloğlu et al., 2004), improved colonic anastomotic healing in a rat experimental sepsis model (Ersoy et al., 2016) and counteracted inducible mitochondrial nitric oxide synthase-dependent mitochondrial dysfunction in skeletal muscle (Escames et al., 2006) and diaphragm (López et al., 2006b) in septic mice.

### Melatonin’s Effect on LPS-Induced Injury

Melatonin has been shown to ameliorate LPS-induced brain injury in neonatal rats (Wong et al., 2014), alleviate LPS-induced placental cellular stress response in mice (Wang et al., 2011) as well as LPS-induced hepatic SREBP-1c activation and lipid accumulation in mice (Chen et al., 2011). Of note, melatonin shown protective effect against BBB damage induced by various stimuli, including transient focal cerebral ischemia in mice (Chen et al., 2006), excitotoxic injury in neonatal rats (Moretti et al., 2015) and methamphetamine-induced inflammation (Jumnongprakhon et al., 2016). Therefore, decreased levels of melatonin in old mice may contribute to the BBB disruption when facing various extrinsic or intrinsic stimuli because melatonin has demonstrated its protective effects against LPS-induced injury to the heart (Lu et al., 2015), brain (Carloni et al., 2016), lung (Lee et al., 2009) and liver (Wang et al., 2007) by scavenging a variety of free radicals (Manchester et al., 2015). Interestingly, chronic melatonin treatment has also shown reduction of age-dependent inflammatory process in senescence-accelerated mice (Rodríguez et al., 2007). In a recent study, Wang et al. (2017) showed that 1 week melatonin treatment significantly alleviated LPS-induced BBB damage accompanied by reduction of occludin and claudin-5 degradation, suggesting that melatonin supplementation is important in decreasing sepsis and neuroinflammation-induced TJPs degradation as well as BBB damage.

### Possible Molecular Mechanism Underlying Melatonin’s Effect on LPS-Induced BBB Damage in Old Mice

Melatonin has shown protective effect on BBB integrity via a variety of pathways: inhibition of the toll like receptor 4 (TLR4)/NF-κB signaling pathway in neonatal rats (Hu et al., 2017), inhibition of NADPH oxidase-2 (Jumnongprakhon et al., 2016), inhibition of MMP-9 (Alluri et al., 2016), inhibiton of AMP-activated protein kinase (AMPK) activation (Wang et al., 2017) and impact on silent information regulator 1 (SIRT1; Zhao et al., 2015) and NLRP3 inflammasome (Rahim et al., 2017).

### AMPK Activation

**Figure 2:**
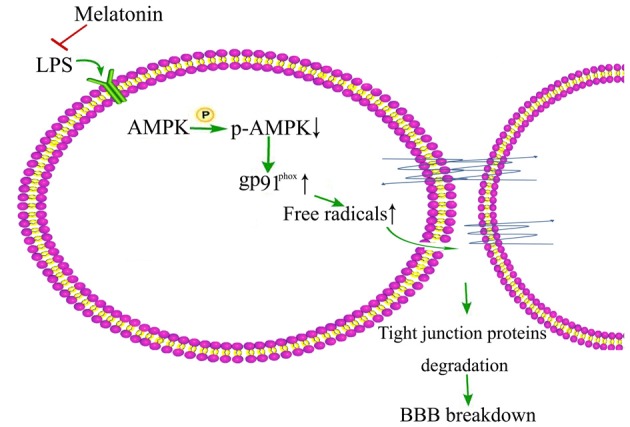
**Schematic illustration of melation’s protective effect on lipopolysaccharide (LPS)-induced damage of BBB integrity**.

AMPK activation has been shown to play important role in maintaining the integrity of BBB (Liu et al., 2012) and it is also reported that LPS inhibits the activation of AMPK, a serine/threonine protein kinase regulating cellular and organismal metabolism (Wang et al., 2017). Interestingly, AMPK activation has been shown to alleviate LPS-induced BBB disruption in both *in vitro* cell model (Zhao et al., 2014) and *in vivo* mice model (Zhou X. et al., 2014; Wang et al., 2017). Activation of AMPK also demonstrated protective effect against diabetes-induced BBB damage by inhibiting NADPH oxidase expression upregulation in brain capillary endothelial cells (Liu et al., 2012). In a recent study, Wang et al. (2017) showed that AMPK activation by melatonin reduced LPS-induced BBB damage in old mice and AMPK activation by metformin decreased LPS-induced gp91^phox^ up-regulation in brain capillary endothelial cells (Figure 2). AMPK activation might be important in maintaining the integrity of BBB in old patients and AMPK dysfunction might play a key role in the initiation and progression of neurological disorders in old people. Therefore, activation of AMPK may be a strategy to reduce neurological disorders following sepsis and neuroinflamation-induced BBB damage in old people.

### Matrix Metalloproteinase-9 (MMP-9)

MMP-9 has been shown to play important role in BBB damage (Jin et al., 2013, 2015; Cai et al., 2015) and melatonin has been shown to bind to MMP-9 to act as its endogenous inhibitor. Melatonin treatment provided protection against traumatic brain injury (TBI)-induced BBB hyperpermeability via MMP-9 inhibition (Alluri et al., 2016), indicating its potential as a therapeutic agent for BBB damage.

### Silent Information Regulator 1 (SIRT1)

SIRT1 was reported to be beneficial in sepsis. Using EX527, a SIRT1 inhibitor, the authors figured out that melatonin alleviated BBB damage in mice which subjected to cecal ligation and puncture via SIRT1 to inhibit inflammation, apoptosis and oxidative stress (Zhao et al., 2015).

### NLRP3 Inflammasome

Aging and sepsis triggered NLRP3 inflammasome activation (Volt et al., 2016), which has been shown to be involved in the innate immune response during inflammation (Rahim et al., 2017). Furthermore, NLRP3 inflammasome activation was showed to be associated with the upregulation of apoptotic signaling pathway in various inflammatory diseases (Volt et al., 2016) and melatonin attenuated subarachnoid hemorrhage-induced BBB damage via attenuating the expressions of NLRP3 (Dong et al., 2016).

### Dark Side/Downsides of Melatonin Supplementation

Although acute toxicity of melatonin is extremely low in both animal and human studies, melatonin may still cause minor adverse effects, such as headache, insomnia and nightmares (Malhotra et al., 2004). Based on previous studies, melatonin could be used as a daily supplement to delay or prevent changes associated with age. However, long-term side effect of melatonin has to be tested, because melatonin has been used as a contraceptive for women which could have reproduction alterations by consumption of melatonin (Voordouw et al., 1992). In addition, there was a decrease in sperm motility in male rats (Gwayi and Bernard, 2002), and long-term administration of melatonin inhibited testicular aromatase levels (Luboshitzky et al., 2002). It does not matter to provide old people with daily melatonin to prevent neurological diseases even if these two side effects may happen as they would not have reproduction anymore. Other side effects should be considered, for example, melatonin may accelerate the development of autoimmune conditions (Mattsson et al., 1994), increase atherosclerosis in the aorta in hypercholesterolemic rats (Tailleux et al., 2002) and produce opposite effects in cancer treatment with poorly timed administration (Bartsch and Bartsch, 1981).

### Conclusion

In conclusion, decreased melatonin levels may account for the BBB damage in old people who often face the common stress of sepsis and neuroinflammation. Melation supplementation treatment significantly inhibits such events. Therefore, continuous daily melatonin supplementation may help prevent sepsis and neuroinflammation-related neurological diseases through maintaining the integrity of BBB in old people. Since melatonin has low toxicity profile and high efficacy in many pathophysiological states, it should be more commonly tested/used in the medical and veterinary arenas. Further studies are needed to verify the important significance of daily melatonin supplementation in old people.

## Author Contributions

W-CL, XW, XZ, XC and XJ wrote the manuscript and XC, XJ obtained the funding. XW drew the figures. All authors have approved the final version of this review article.

## Conflict of Interest Statement

The authors declare that the research was conducted in the absence of any commercial or financial relationships that could be construed as a potential conflict of interest.

## Abbreviations

AMPK: AMP-activated protein kinase
BBB: blood brain barrier
CNS: central nervous system
iNOS: inducible nitric oxide synthase
LPS: lipopolysaccharide
MMP-9: matrix metalloproteinase-9
nNOS: neuronal nitric oxide synthase
NADPH: nicotinamide adenine dinucleotide phosphate
NF-κB: nuclear factor kappa-light-chain-enhancer of activated B cells
NLRP3: nucleotide-binding oligomerization domain-like receptor family pyrin domain-containing 3
ROS: reactive oxygen species
SIRT1: silent information regulator 1
TBI: traumatic brain injury
TJPs: tight junction proteins
TLR4: toll like receptor 4.
